# The Abundance of FOXP3, FOXP3/CD4 and CD8 Cells in the Microenvironment of Nodular Sclerosis and Mixed Cellularity Subtypes Is Associated with the Epstein–Barr Virus Status of Classic Hodgkin Lymphoma

**DOI:** 10.3390/biomedicines12081680

**Published:** 2024-07-27

**Authors:** Antonia Pavlović, Antonija Miljak, Katarina Brzica, Merica Glavina Durdov

**Affiliations:** 1 Department of Pathology, Forensic Medicine and Cytology, University Hospital of Split, 21000 Split, Croatia; merigdst@yahoo.co.uk; 2 Department of Hematology, University Hospital of Split, 21000 Split, Croatia; antonija.miljak003@gmail.com; 3 Department of Pediatric, University Hospital of Split, 21000 Split, Croatia; katarina.brzica00@gmail.com

**Keywords:** Hodgkin lymphoma, subtypes, T regulatory cells, FOXP3, EBV

## Abstract

Thymic regulatory lymphocytes (Tregs) are rare in the normal periphery where they mediate immune tolerance but accumulate in the tumor immune microenvironment (TIM), reducing the antitumor response. Subtypes of classical Hodgkin lymphoma (CHL) are characterized by a minority of malignant Hodgkin and Reed–Sternberg cells (HRS) and an abundant TIM that plays a key role in modulating the disease. CHL is related to the Epstein–Barr virus (EBV), whose oncogenes influence the growth of HRS. We analyzed the number of T lymphocytes expressing the regulatory marker FOXP3 in CHL with regard to EBV status. The tumor tissue of 182 patients was stained by double immunohistochemistry for FOXP3, CD4, and CD8, and the number of different phenotypes was analyzed microscopically. EBV status was determined by EBER in situ hybridization. EBV-positive CHL was confirmed in 28% of patients and was associated with mixed cellularity (MC) (*p* < 0.001), older age (*p* < 0.001), and unfavorable outcomes (*p* = 0.038). The number of CD8+ T lymphocytes differed according to the EBV status of MC and nodular sclerosis (NS), and was the lowest in EBV-negative NS (*p* = 0.001). Likewise, the numbers for FOXP3 and FOXP3/CD4 were different, and were the lowest in EBV-negative MC (*p* = 0.035 and *p* = 0.041, respectively). Values above a median of FOXP3 and CD4 are associated with longer progression-free survival (*p* = 0.039 and *p* < 0.001, respectively). EBV impacts the composition of T cell phenotypes in TIM, among which the amount of CD4 and FOXP3 is prognostically valuable.

## 1. Introduction

Hodgkin lymphoma (HL) is one of the most common lymphomas in the Western world. It is a B-cell neoplasm characterized histologically by a few malignant Hodgkin and Reed–Sternberg (HRS) cells in an abundant but anergic tumor immune microenvironment (TIM) [1]. Based on the morphology, immunophenotype of HRS cells, and clinical behavior, HL is divided into lymphocyte-predominant nodular HL and classic HL (CHL), the latter consequently split into four distinct subtypes: nodular sclerosis (ND), mixed cellularity (MC), lymphocyte rich (LR), and lymphocytes depleted (LD) [2]. Epstein–Barr virus (EBV) is present in HRS in approximately 40% of cases [3]. Viral oncoproteins and small non-coding RNAs (EBV-encoded RNA, EBER) cause downregulation of B cell phenotype, cell proliferation, and affect TIM composition [4,5]. Although the content of TIM varies among different CHL subtypes, T lymphocytes, mainly CD4 helper lymphocytes, usually predominate. Numerous immune cells are unable to eliminate rare malignant HRS cells, which, according to the article by Coosman et al. from 1998, “survive in a hostile sea” [6]. The discovery of regulatory T cells (Tregs) helped us to understand the anergic TIM in CHL [7]. Tregs are a special cell population responsible for maintaining self-tolerance and an appropriate immune response by controlling the proliferation and activation of T lymphocytes, B cells, NK cells, and antigen-presenting cells [8,9]. Natural T regulatory cells originate from the thymus and express the transcription factor FOXP3 (forkhead box P3), and the markers Helios and Neuropilin-1 [10]. During maturation in the thymus, in the case of high-affinity antigen recognition with CD28/CD80 co-stimulation, a strong expression of the IL-2 receptor (CD25) is induced [11]. The interaction of CD25 and IL-2 activates signaling pathways and JAK-STAT kinase to regulate the FOXP3 promoter region toward a regulatory phenotype. Even before birth, mature Tregs enter peripheral blood and constitute 5–10% of CD4 T lymphocytes in healthy individuals. A smaller number of adaptive Tregs, which are FOXP3 negative, develop in the periphery from naïve T lymphocytes after IL-10 and TGF-β stimulation, and with appropriate epigenetic DNA modification [12,13,14]. Long-term Treg proliferation depends on IL-2 from surrounding effector cells [15]. Patients with malignant disease have significantly more Tregs in peripheral blood and TIM than healthy controls [16,17]. Unlike most solid tumors, a higher proportion of Tregs in the TIM of head and neck tumors, indolent NHL, and CHL is associated with a better prognosis [18,19]. In CHL, chemokines secreted by HRS selectively attract Tregs from the blood to the TIM, where they bind to ligands on tumor/stromal cells and contribute to the antitumor anergic response [20,21]. In addition, in EBV-positive CHL, EBV latent antigens modulate the tumor milieu with a heterogeneous population of FOXP3 Treg lymphocytes [22]. We previously found significantly more FOXP3 regulatory cells in the TIM of EBV-associated CHL [23]. In this larger study, we analyzed the amount of FOXP3, CD4, and CD8 in subtypes of CHL according to EBV status and impact on outcome.

## 2. Materials and Method

### 2.1. Patients and Data Collection 

This research included 182 patients with CHL consecutively diagnosed at the Department of Pathology, Forensic Medicine and Cytology, University Hospital of Split, Croatia from 1 January 1997 to 31 December 2018. Paraffin blocks of tumor tissue were collected from the department, and clinical data from the hospital records of the Department of Hematology and the Department of Pediatrics. Patients were treated according to the ABVD protocol (adriamycin, bleomycin, vinblastine, dacarbazine), with additional radiotherapy in the case of bulky disease or local residual tumor. The escalated e-BEACOPP protocol (escalated doses of bleomycin, etoposide, doxorubicin, cyclophosphamide, vincristine, procarbazine, and prednisone) was established in 2017 for advanced-stage CHL.

Relapse of the disease was proven by imaging methods (CT, PET/CT) and/or morphologically, by fine needle aspiration or biopsy of the available lymph node. We received information regarding the deaths from the coroner’s office of Split-Dalmatia County. Exclusion criteria were the lack of a paraffin block of tumor tissue and incomplete clinical data. Follow-up began from the date of pathologic diagnosis and continued until the date of the last clinical follow-up or death, ending on 5 June 2023, when the patient’s survival status was determined as alive or dead. Median follow-up was 132 (min–max, 1–285 months). Progression-free survival (PFS) and overall survival (OS) were calculated in months. Input parameters were age, gender, subtype, B symptoms and stage. Outcome parameters were CD4, CD8, FOXP3, FOXP3/CD4, and FOXP3/CD8 phenotype T cell counts in TIM, as well as survival status, PFS and OS.

### 2.2. Methods 

Histological slides 3 μm in thickness were cut from paraffin blocks of tumor tissue and mounted on silanized glass. Staining was performed automatically on the Ventana Ultra Benchmark (Ventana Roche, Tucson, AZ, USA). Anti-CD4 rabbit monoclonal antibody (clone SP35, Ventana Roche, Tucson, AZ, USA) or anti-CD8 rabbit monoclonal antibody (clone SP57, Ventana Roche, Tucson, AZ, USA) was co-applied with mouse monoclonal antibody FOXP3 (clone 236A/E7, Santa Cruz Biotechnology, Dallas, TX, USA) diluted 1:50 and incubated overnight. For detection were used Ultraview DAB detection kit and UltraView Universal Alkaline Phosphatase Red Detection Kit (both Ventana Roche, Tucson, AZ, USA). A positive reaction was red membranous/cytoplasmatic staining for CD4 or CD8 and brown nuclear staining for FOXP3.

**Figure 1:**
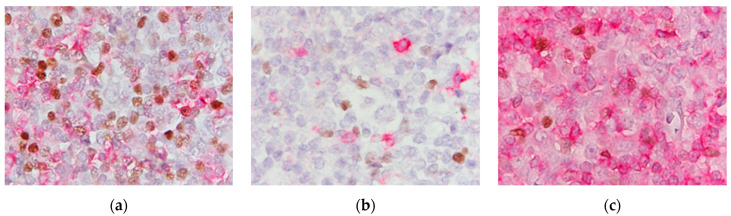
Analyzed phenotypes of T cells in tumor microenvironment according to EBV status of CHL. (**a**) Numerous FOXP3 and CD8 cells and some FOXP3/CD8 cells in EBV-positive nodular sclerosis; (**b**) paucity of FOX3, CD8 and FOXP3/CD8 cells around HRS in EBV-negative nodular sclerosis; (**c**) plenty of FOXP3/CD4+ cells and CD4 T cells in EBV-positive mixed cellularity (FOXP3 brown nuclei, CD8 and CD4 red cytoplasm, 1000×).

Slides were analyzed on an Olympus BX46 microscope (Olympus, Tokyo, Japan) in 10 randomly selected non-overlapping fields of high magnification, at 1000× (field width 0.1 mm), which was equivalent to a tumor area of 1 mm^2^. Only the fields with the presence of HRS cells in the tumor microenvironment were analyzed, while the peripheral parts of the lymph node were ignored. We simultaneously microscopied and counted the different phenotypes using a differential blood cell counter (Diffcount III, Modulus Data Systems, inc., New York, NY, USA). Thus, the total sum of each analyzed phenotype in 1 mm^2^ was obtained for that patient. Later, the slides were photographed with a digital camera Olympus DP26 (Olympus, Tokyo, Japan) in ten randomly selected non-overlapping fields at magnification 1000× for documentation. Thus, cell counting was done by looking at the field of view on the microscope, while the expert (first author) counted the cells with her left hand using a cell counter. Counting was not done from the captured image. The captured image was included in the Figure 1 so that readers can have insight into the section.

EBER in situ hybridization was performed automatically on the Ventana Ultra Benchmark system using an EBER oligoprobe (INFORM EBER Probe, Ventana Roche, Tucson, AZ, USA) and a detection system (ISH iView Blue Detection Kit, Ventana Roche, Tucson, AZ, USA). The positive reaction was black-blue nuclear staining of HRS cells, and the positive external control was EBV-positive nasopharyngeal carcinoma. The negative control was a histological slide in which the EBER probe was omitted in the staining process. A clear positive nuclear EBER signal in one or more malignant HRS cells was evidence of the presence of EBV and the tumor was determined as EBV-positive CHL.

### 2.3. Statistical Analysis

Statistical Package for the Social Sciences (SPSS) software (version 19 for Windows; SPSS Inc., Chicago, IL, USA) was applied for statistical analysis. Qualitative variables were described as absolute numbers and percentages. Medians, lower and upper quartiles and min–max values were used to describe the distribution of quantitative variables. For normally distributed continuous variables, the *t*-test was applied, and for non-normally distributed continuous variables, the non-parametric Mann–Whitney U test and Kruskal–Walls with pairwise post hoc was used. The χ^2^ test was used to compare categorized variables. The correlation was assessed by Pearson’s correlation coefficient. The Kaplan–Meier curve and log-rank test were used to analyze PFS and OS, as well as Cox univariate and multivariate regression analysis and ROC analysis. The level of significance of the difference was ≤0.05.

### 2.4. Ethical Approval

This study met ethical standards of the Institutional and National research committee and the 1964 Declaration of Helsinki and its subsequent amendments or comparable ethical standards. The Institutional Review Board of the University Hospital of Split approved the study (500-03/19-01/77; date of approval: 30 September 2019).

## 3. Results

### 3.1. Descriptive Data

The sample included 182 patients with CHL, including 86 (47%) women and 96 (53%) men, aged 5 to 87 years, with an average age of 39 ± 19.16 years, median 35, range 5–87 years.

Table 1 presents the value of T-cell phenotypes in the microenvironment of CHL. The most common were CD4 T lymphocytes and the rarest were FOXP3/CD8 lymphocytes.

The distribution of patients according to clinical parameters is shown in Table 2. The most common were the NS and MC subtypes. In total, 51 (28%) patients had CHL associated with EBV. During the follow-up period, 28 patients relapsed and 36 died.

### 3.2. Clinical and Pathological Parameters According to EBV Status of CHL

Patients with EBV-positive CHL were 20.5 (95% CI 12.5–35) years older than patients with EBV-negative CHL (Z = 4.8; *p* < 0.001) (Table 3). The distribution according to subtype was also different in relation to the EBV status of CHL (χ^2^ = 42; *p* < 0.001). In the EBV-positive CHL group, there were no patients with the LR subtype. The proportion of patients with NS in the group of EBV-negative CHL was 2 times higher than in the group of EBV-positive CHL, and the proportion of patients with MC was 4.3 times higher in the group of EBV-positive CHL than in the group of EBV-negative CHL. The distribution of patients according to the stage of the disease differed in relation to the EBV status of CHL at a significance level of 91% (χ^2^ = 6.5; *p* = 0.089). As stage I of the disease was analyzed in relation to the others (II, III and IV), a statistically significant association of stage I with EBV-positive status was confirmed (χ^2^ = 4.5; *p* = 0.033). The distribution of patients according to survival status (alive/dead) was different in relation to EBV status (χ^2^ = 6.5; *p* = 0.038). The proportion of deceased in EBV-positive HL was twice as high as in EBV- negative CHL. The FOXP3+ cell median was 268 (95% CI 38–471), higher in EBV-positive CHL than in EBV-negative CHL (Z = 1.95; *p* = 0.049). The CD8+ T cell median was 257 (95% CI 69–372), higher in EBV-positive CHL than in EBV negative CHL (Z = 3.33; *p* = 0.001). The EBV status of CHL was not connected with FOXP3 + /CD8+ T cells (Z = 1.30; *p* = 0.193), CD4+ T cells (Z = 0.158; *p* = 0.875), or FOXP3/CD4 cells (Z = 1.64; *p* = 0.100).

### 3.3. Clinical and Pathological Parameters According to EBV Status of Common Subtypes

NS and MC, as the most common subtypes of CHL, were compared, and it was found that the number of CD8+ T lymphocytes differs with regard to EBV status (χ^2^ = 19.1; *p* < 0.001) (Table 4). In EBV-negative NS there were less CD8+ T lymphocytes than in EBV-positive NS (*p* < 0.01), in EBV-negative MC (*p* = 0.035) and EBV-positive MC (*p* = 0.026). Likewise, the number of FOXP3 cells was different in NS and MC with regard to EBV status (χ^2^ = 8.6; *p* = 0.035). In EBV-negative MC there were significantly less FOXP3 cells than in EBV-positive MC (*p* = 0.004), EBV-negative NS (*p* = 0.034), or EBV-positive NS (*p* = 0.039). In addition, the number of FOXP3/CD4 T cells was significantly different in NS and MC respecting the EBV status (χ^2^ = 8.2; *p* = 0.041), being less in EBV-negative MC than in EBV-positive MC (*p* = 0.004), as well in EBV-negative NS (*p* = 0.034) and EBV-positive NS (*p* = 0.073). Between NS and MC, regarding EBV status, there was no significant difference in the number of CD4 + T lymphocytes (χ^2^ = 0.551; *p* = 0.908) and FOXP3/CD8 T lymphocytes (χ^2^ = 1.8; *p* = 0.607).

In EBV-positive CHL there was a higher amount of FOXP3 cells, CD8 and FOXP3/CD4 cells. Figure 1 shows photomicrographs with a different number of analyzed phenotypes in the tumor immune microenvironment of NS and MC according to EBV status.

### 3.4. Clinical and Pathological Parameters According to Relapse

On average, 28 patients had a relapse of disease (Table 5). There was correlation of relapse with B symptoms (χ^2^ = 5.8; *p* = 0.016) and survival status (χ^2^ = 17.7; *p* < 0.001). The risk of death was 3.4 times higher (95% CI 1.3–8.8; *p* = 0.011) in the group of patients with B symptoms compared to patients without B symptoms and 10.3 times higher (95% CI: 3.2–32; *p* < 0.001) in patients with relapse than in patients without relapse. There was no correlation of relapse with gender (χ^2^ = 0.038; *p* = 0.846), subtype (χ^2^ = 1.76; *p* = 0.416), EBV status (χ^2^ = 0.001; *p* = 0.978), and stage (χ^2^ = 5.6; *p* = 0.134). Analyzed phenotypes of T cells were not associated with relapse: FOXP3 (Z = 1.1; *p* = 0.268), CD8 (Z = 0.266; *p* = 0790), FOXP3/CD8 (Z = 0.329; *p* = 0.742), CD4 (Z = 1.68; *p* = 0.94), and FOXP3/CD4 (Z = 0.628; *p* = 0.530).

Multiple Cox regression analysis, in which FOX and CD4 were included, has confirmed the association between the amount of CD4 in the TIM and the occurrence of relapse (Table 6).

### 3.5. Clinical and Pathological Parameters According to B Symptoms

The correlation of analyzed variables with B symptoms is shown in Table 7. B symptoms were associated with subtype (χ^2^ = 6.6; *p* = 0.037), stage (χ^2^ = 22; *p* < 0.037), survival status (χ^2^ = 4.1; *p* = 0.043), and CD4 phenotype (Z = 2.4: *p* = 0.017). There was no correlation with age (Z = 0.046; *p* = 0.963), EBV status (χ^2^ = 1.15; *p* = 0.282), FOXP3 (Z = 0.322; *p* = 0.747), CD8 (Z = 1.84; *p* = 0.066), FOXP3/CD8 (Z = 0.116; *p* = 0.246), or FOXP3/CD4 (Z = 0.020; *p* = 0.984).

The proportion of deceased patients was 2.3 times higher in the group of patients with B symptoms than in the group without B symptoms. The proportion of patients with disease stage I was 7 times higher in the group without B symptoms compared to the group with B symptoms. The proportion of patients with MC was 2.2 times higher in the group without B symptoms than in the group with B symptoms. The proportion of patients with stage III was 2.3 times higher in the group with B symptoms than in the group without B symptoms. The proportion of patients with stage IV was 2.1 times higher in the group with B symptoms than in the group without symptoms. The median of CD4+ cells was 553 higher in the group without B symptoms than in the group with B symptoms.

During the follow-up period, 155 patients did not relapse, and 28 patients relapsed, with a median PFS of 8 (min–max, 1–63) months. We analyzed all variables according to PFS of all patients (Table 8).

**Figure 2:**
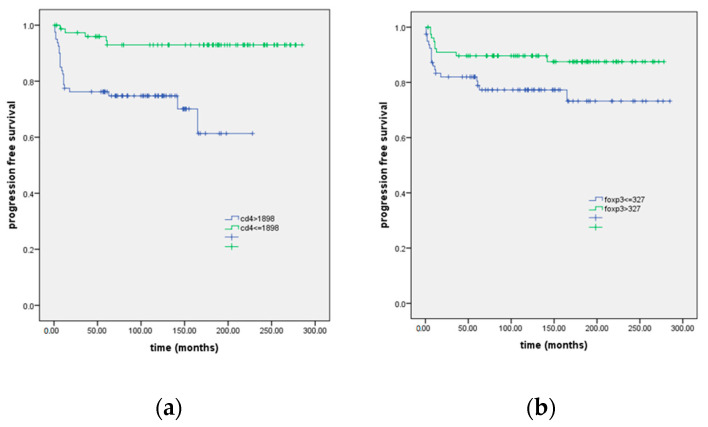
Kaplan–Meier curve of progression-free survival in the most common subtypes of classical Hodgkin lymphoma according to values lower and higher than the median of CD4 (**a**) and FOXP3 (**b**).

CD4 and FOXP3 were significantly associated with progression-free survival. Hazard ratio was 5.5 times lower in patients with CD4 ≤ 1899 than in patients with CD4 >1899 (*p* = 0.001). Likewise, hazard ratio was 2.3 times lower in patients with FOXP3 ≤ 327 than in patients with FOXP3 >327 (*p* = 0.046). Gender, age, subtype, B symptoms, and stage, as well as median CD8, FOXP3/CD8, and FOXP3/CD4 were not correlated with PFS. Figure 2 shows the association of CD4 and FOXP3 phenotypes with progression-free survival. Patients with CD4 ≤1899 had PFS 163.5 (11,809) 140.4–186.7 months, and patients with CD4 >1899 had PFS 267.4 (7.5) 252.6–282.3 months (log rank 14,079; *p* > 0.001).

**Figure 3:**
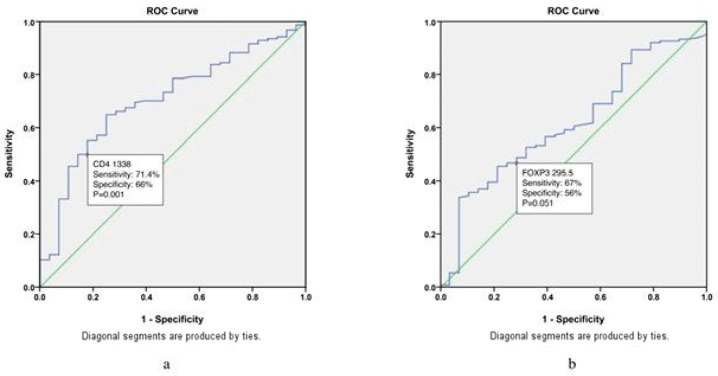
Cutoff values of 1338 for CD4 (**a**) and 295.5 for FOXP3 (**b**) distinguish patients with and without relapsed classical Hodgkin lymphoma

ROC analysis was performed to determine the threshold value of CD4 and FOXP3 for determining patients with and without relapse (Figure 3).

The cutoff value of CD4 for delineating patients with and without relapse was 1338, with a sensitivity of 71.4% and specificity of 66% (area: 0.705; SE:0.049; 95% CI 0.608–0.802; *p* = 0.001). The cutoff value of FOXP3 for delineating patients with and without relapse was 295.5 with a sensitivity of 60.7% and specificity of 56% (area: 0.616; SE 0.054; 95% CI 0.509–0.722; *p* = 0.051).

OS was 158 (5.6); 147–169 months. On average, 36 patients died. PFS in the group of alive patients was 95 months longer than in the group of those deceased (LR 10.2; *p* = 0.006). Alive patients without B symptoms had 64 months longer PFS than patients without B symptoms (LR 6.8; *p* = 0.009). The association of relapse to OS is shown in Table 9.

Patients who relapsed had shorter OS (LR 26.6; *p* > 0.001).

## 4. Discussion

In classic Hodgkin lymphoma, HRS cells are intertwined with the immune microenvironment in many ways. TIM provides survival signals to HRS, while HRS cells modulate TIM composition. HRS cells create an anergic, immunosuppressive microenvironment by complex and diverse mechanisms [24]. Partly responsible for this peculiar specificity in CHL are the Treg cells in the TIM, which are the focus of this research. In our study of 182 patients from a single regional institution, 51 (28%) had EBV-associated CHL, similar to our previous studies and studies in the Mediterranean region [23,25,26]. Patients with EBV-positive CHL were 20.5 years older than patients with EBV-negative CHL, and 62% of MC subtype cases were EBV-positive. Both results are in accordance with the literature [2]. EBV infection is ubiquitous and lifelong in humans; due to immunosenescence, the immune system gradually loses control over EBV latently infected B-lymphocytes [3]. The MC subtype is more common in deprivileged societies worldwide, and is often associated with EBV. In the region of southern Croatia, a decrease in the MC subtype from 37% (patients diagnosed in 1980–1990) to 24.2% (patients diagnosed in 1997–2018) and a simultaneous increase in NS from 48 to 68.1% can be observed [23,25]. This could suggest a better socioeconomic standard for our population. EBV-positive CHL was diagnosed more often in stage I compared to other stages. EBV-associated CHL was more common in patients with a fatal outcome.

Median of FOXP3 was higher in EBV-positive CHL than in EBV-negative CHL, which is consistent with our previous study and those of other authors [23,27]. The median of CD8+ T cells was significantly higher in EBV-positive NS than in EBV-negative NS, as well as in MC regardless of EBV status. On the other hand, the amount of FOXP3 and FOXP3/CD4 was lower in MC-negative CHL than in EBV-positive MC and NS regardless of EBV status. This difference highlights the complex pathophysiological mechanism that creates the histological subtypes [2]. Assiz et al. found that increased expression of CD4 + CD25 + FOXP3+ regulatory T cells correlated with the EBV status of CHL, but had no effect on survival [28]. Alvaro et al. found that a high infiltration of cytotoxic cells combined with low infiltration of FOXP3 regulatory cells could predict adverse outcomes [29]. In this study, we confirmed the beneficial effect of CD4 and FOXP3, as values higher than their median were associated with longer PFS.

Although Treg cells in CHL are mainly of thymic origin (FOXP3 positive), FOXP3 negative Treg cells also exist in the tumor microenvironment. Morales et al. showed that these cells produce IL-10 due to the effect of EBV protein on gene transcription [22]. Thus, FOXP3-positive Treg cells in CHL represent the “tip of the iceberg” of the population of regulatory cells that contribute to immunosuppression in CHL and make this tumor different from others. HRS cells express CCL17, CCL22 and MIP3α which attract Treg cells to their microenvironment [24]. According to Baumforth et al., in EBV-associated CHL, EBNA1 leads to CCL20 expression on the HRS and increased migration of FOXP3-positive Treg cells into the microenvironment [30]. The authors showed a direct effect of EBV on the number of FOXP3+ Treg cells in CHL, but found no difference in Treg number between EBV-positive and EBV-negative cases, possibly due to the small sample size of 23 patients and three cell lines.

In our sample, the lower number of FOXP3/CD4 and FOXP3 cells in EBV-negative MCs can be explained by the absence of an effect of EBNA1 on Treg migration in TIM. Cytotoxic CD8 lymphocytes are generally less abundant in the CHL microenvironment than CD4 lymphocytes. Meij et al. found that CD8+ cells were directed against the EBV-encoded oncoproteins LMP1 and LMP2 [31]. Early lytic EBV proteins are potent stimulators of the cytotoxic T-cell response, for example in infectious mononucleosis, but viral proteins in latent infection or EBV-positive tumors are much less stimulating of the cytotoxic response. In EBV-positive CHL, only part of the viral EBNA1 is present on MHC I [5]. A microenvironment rich in Th2 helper lymphocytes secretes cytokines that suppress the CD8+ T cell response [32]. HRS cells and CD8 T cells secrete IL-10 and TGFβ that trigger the development of adaptive Treg lymphocytes [33]. HRS cells develop resistance to CD8 lymphocyte destruction, mediated by granzyme and perforin. Mechanisms controlled by HRS cells and different subsets of immune cells form an immunosuppressive network against an effective immune response [34]

Kiyasu et al. analyzed HIV-positive immunosuppressed patients with EBV-positive CHL. These patients had fewer FOXP3-positive cells and more CD8 lymphocytes than immunocompetent HIV-negative patients with CHL [35]. Controls with EBV-positive HL had higher numbers of FOXP3 cells and CD8 cells than EBV-negative HL, which is similar to the results in our study. Kiyasu et al. believe that the CD8 T response does not represent LMP1-specific CD8 T lymphocytes, but anergic T cells. The increased number of CD8 cells in EBV-associated HL is probably not an antiviral response, but rather a population of FOXP3-negative regulatory cells that do not produce granzyme or perforin and are therefore ineffective. In our study, the population of CD8 regulatory lymphocytes was certainly “underestimated” using FOXP3 alone, and more markers are needed to determine their contribution to the CHL microenvironment.

Treg cells in the TIM are partially responsible for the anergic antitumor response. Constitutively activated signaling pathways and/or genetic dysregulation in HRS lead to low expression of MHC I and high expression of the checkpoint ligand PD-L1. The interaction between PD-L1 on HRS cells and PD-1 on a specific T lymphocyte inhibits its proliferation and effector function, leading to immune evasion [36,37,38,39]. Deregulation of the PD-L1/PD-1 axis is present in EBV-positive and EBV-negative CHL, but the effect is achieved by slightly different mechanisms. In EBV-negative CHL, frequent chromosomal changes lead to overexpression of PD-L1 and PD-L2, and abundant surrounding lymphocytes are “perfect targets to block”. In EBV-positive CHL, aberrantly activated JAK-STAT signaling pathways in HRS cells enhance promoter regions for the PD-L1/PD-1 axis with the same result. This scenario could be responsible for TIM anergy in cases of CHL with a low number of FOXP3 cells, acting as an alternative mechanism for inadequate antitumor response.

The strengths of this study are as follows: this is a real-life study from a single institution, performed on whole paraffin blocks of tumor tissue and the number of patients was relatively large. The disadvantage of this retrograde study is that a compatible flow cytometry method could not be applied.

## 5. Conclusions

EBV has an impact on the composition of the T cell population in CHL, which is characterized by more CD8, FOXP3 and FOXP3/CD4 T cells. Values above median of FOXP3 and CD4 are associated with longer progression-free survival. Threshold values of 1338 of CD4 and 295.5 of FOXP3 could be used for the delineation of patients with/without relapse. These results need to be validated in a larger sample, in order for them to become potentially clinically useful histological markers for predicting relapse of CHL.

## Figures and Tables

**Table 1:** Phenotypes of T cells in the microenvironment of classic Hodgkin lymphoma counted on 10 fields of the highest magnification (1000×).

Variable	Mean (SD)	Median (Q1–Q3) Min–Max
FOXP3	468 ± 428	327 (Q1–Q3) 153–707 min–max 17–2368
CD8	555 ± 434	435 (Q1–Q3) 266–707 min–max 33–2899
FOXP3/CD8	23.9 ± 51	8 (Q1–Q3) 2–24 min–max 0–431
CD4	2037 ± 1141	1898 (Q1–Q3) 995–2962 min–max 4–5318
FOXP3/CD4	404 ± 402	263 (Q1–Q3) 110–599 min–max 16–2355

SE: standard deviation, Q1: lower quartile, Q3: upper quartile, min: minimal, max: maximal.

**Table 2:** Number (%) of patients with classic Hodgkin lymphoma according to clinical parameters.

Variable		N (%)
Gender	female	86 (47)
	male	96 (53)
Subtype	nodular sclerosis	124 (68.1)
	mixed cellularity	44 (24.2)
	lymphocyte rich	8 (4.4)
	lymphocyte depleted	6 (3.3)
Epstein–Barr virus	positive	51 (28)
	negative	131 (72)
Stage	I	21 (13.8)
	II	70 (46.1)
	III	37 (24.3)
	IV	24 (15.8)
B symptoms	no	72 (46.7)
	yes	82 (53.2)
Relapse	no	96 (52.7)
	yes	28 (15.4)
	unknown	58 (31.9)
Survival status	alive	116 (63.7)
	dead	36 (19.8)
	unknown	30 (16.6)

**Table 3:** Number (%) of patients according to qualitative variables and median (Q1–Q3; min–max) of quantitative variables according to EBV status of classic Hodgkin lymphoma.

		Classic Hodgkin Lymphoma		
Variable		EBV-Positive	EBV-Negative	*p* *
		N(%) or Median (Q1–Q3) Min–Max		
Gender	female	22 (43)	64 (49)	0.631 *
	male	29 (57)	67 (51)	
Age	years	54.5 (37–68; 6–87)	31 (21–46; 5–86)	<0.001 **
Subtype	NS	20 (39.3)	104 (80)	<0.001 *
	MC	27 (52.9)	16 (12.3)	
	LR + LD	4 (7.8)	10 (10.7)	
B symptoms	no	21 (55)	50 (43)	0.282 *
	yes	17 (44)	65 (57)	
Stage	I	9 (24.3)	12 (10.4)	0.089 *
	II	13 (35.1)	57 (49.6)	
	III	11(29.7)	26 (22.6)	
	IV	4 (10.8)	20 (17.4)	
Survival status	alive	26 (51)	89 (68.5)	0.038 *
	dead	16 (31.4)	20 (15.4)	
	unknown	9 (17.6)	21 (16.2)	
FOXP3		560(183–902; 22–1413)	292(150–536; 17–2368)	0.049 **
CD8		620(386–893; 33–2899)	363(246–603; 55–1509)	0.001 **
FOXP3/CD8		12(2.7–37; 0–280)	7(2–20; 0–431)	0.193 **
CD4		2009(968–2919; 587–4221)	1802(995–3053; 4–5318)	0.875 **
FOXP3/CD4		348.5(115–772; 16–1347)	223(109–480; 16–2355)	0.1 **

* χ^2^ test, ** Mann–Whitney U test. Legend: EBV Epstein–Barr virus; Q1, lower quartile; Q3, upper quartile; NS, nodular sclerosis; MC, mixed cellularity; LR, lymphocyte rich; LD, lymphocyte depleted.

**Table 4:** Values (median and range) of analyzed T cell phenotypes in the tumor microenvironment in common subtypes of classic Hodgkin lymphoma according to EBV status.

	Classic Hodgkin Lymphoma					
	EBV-Positive		EBV-Negative			
Phenotype	NS	MC	NS	MC	χ^2^	p *
	Median (Range)					
CD8	700 (160–2899)	500 (33–2600)	336 (55–1509)	558 (142–1433)	19.1	0.001
CD4	1893 (587–4221)	1994 (859–4100)	1727 (4–5318)	1402 (779–4946)	0.551	0.908
FOXP3	329 (22–412)	573 (39–1324)	316 (19–368)	163 (17–982)	8.6	0.035
FOXP3/CD8	13 (0–218)	10 (0–117)	7 (0–431)	13 (0–57)	1.8	0.607
FOXP3/CD4	270 (16–1347)	558 (35–1144)	273 (18–2355)	143 (16–878)	8.2	0.041

* Kruskal–Wallis test. Legend: EBV Epstein–Barr virus NS nodular sclerosis MC mixed cellularity.

**Table 5:** Number (%) of patients with classic Hodgkin lymphoma according to qualitative and quantitative variables (median (Q1–Q3; min–max) in relation to relapse.

	Relapse			
Variable	No (N = 96)	Yes (N = 28)	*p*	OR (95% CI); *p* ***
	N(%) or Median (Q1–Q3) Min–Max			
Gender			0.846 *	
female	46 (48)	14 (50)		
male	50 (52)	14 (50)		
Age	32 (22–50; 10–83)	33 (20–49; 13–86)	0.894 **	
Subtype			0.416 *	
NS	67 (69.8)	23 (82.1)		
MC	25 (26)	4 (14.3)		
LR + LD	4 (4.2)	1 (3.6)		
EBV status-			0.978 *	
positive	24 (25)	7 (25)		
negative	71 (75)	21 (75)		
Stage			0.134 *	
I	15 (16)	0		
II	44 (46.8)	15 (53.6)		
III	18 (19.1)1	8 (28.6)		
IV	7 (18.1)	5 (17.9)		
B symptoms			0.016 *	3.4 (95%CI: 1.3–8.8); *p* = 0.011 ***
no	50 (53.2)	7 (25)		
yes	44 (46.8)	21 (75)		
Survival status			<0.001 *	10.3 (95% CI: 3.2–32); *p* < 0.001 ***
alive	84 (87.5)	15 (53.6)		
dead	6 (6.2)	11 (39.3)		
unknown	6	2		
FOXP3	299 (124–627; 17–1960)	263 (82–379; 40–1560)	0.268 **	
CD8	362 (246–542; 33–2899)	342 (245–531; 167–1509)	0.790 **	
FOXP3/CD8	12 (5–27; 0–431)	10 (14–23; 1–92)	0.742 **	
CD4	1385 (946–2381; 4–4946)	1006 (871–1399; 377–3691)	0.94 **	
FOXP3/CD4	202 (84.494; 16–1514)	188 (72–331; 19–1564)	0.53 **	

* χ^2^ test, ** Mann–Whitney U test, *** logistic regression. Legend: NS, nodular sclerosis; MC, mixed cellularity; LR, lymphocyte rich; LD, lymphocyte depleted; Q1, lower quartile; Q3, upper quartile; CI, confidence interval.

**Table 6:** Results of multiple Cox regression.

Variable	HR(95% CI); *p* *
CD4	4.8 (1.7–13); 0.002
FOXP3	1.5 (0.7–3.5); 0.312

* multiple regression analysis Legend: HR, hazard ratio; CI, confidence interval.

**Table 7:** Number (%) of patients with classic Hodgkin lymphoma according to qualitative and quantitative variables (median (Q1–Q3; min–max) in relation to B symptoms.

Variable	B Symptoms		
	No (N = 72)	Yes (N = 82)	*p*
	N(%) or Median (Q1–Q3) Min–Max		
Gender			0.258 *
female	40 (56)	37 (45)	
male	32 (44)	45 (55)	
Age (years)	35 (24–51; 5–78)	35 (22–50; 13–86)	0.963 **
Subtype			0.037 *
NS	45 (62.5)	65 (79,3)	
MC	23 (31.9)	12 (14.6)	
Epstein–Barr virus			0.282 *
positive	21 (30)	17 (21)	
negative	50 (70)	65 (79)	
Stage			<0.001 *
I	18 (25.4)	3 (3.7)	
II	36 (25.7)	35 (42.7)	
III	10 (14.1)	27 (32.9)	
IV	7 (9.9)	17 (20.7)	
Survival status			0.043 *
alive	57 (88)	49 (72)	
dead	8 (12)	19 (28)	
FOXP3	310 (138–795; 17–1960)	327 (137–548; 22–1560)	0.747 **
CD8	459 (277–687; 55–2899)	346 (243–593; 33–1722)	0.066 **
FOXP3/CD8	12.5 (3–28; 0–431)	8 (2–22; 0–126)	0.246 **
CD4	1899 (1099–3048;5 81–5318)	1346 (867–2510; 4–4241)	0.017 **
FOXP3/CD4	209 (101–599; 16–1514)	274 (108–528; 16–1564)	0.984 **

* χ^2^ test, ** Mann Whitney U test, Legend: CHL, classic Hodgkin lymphoma; NS, nodular sclerosis; MC, mixed cellularity; Q1, lower quartile; Q3, upper quartile; min, minimum; max, maximum.

**Table 8:** Progression free survival of patients with classic Hodgkin lymphoma in relation to the analyzed variables.

	Progression Free Survival (Months)		
Variable	Mean (SE; 95%CI)	LR; *p*	HR (95% CI); *p* *
Gender		0; 0.998	
female	237 (11; 1; 215–260)		
male	232 (12; 209–255)		
Age		0.115; 0.734	
≤35 years	236 (11; 213–258)		
>35 years	230 (11; 208–252)		
Subtype		1.8; 0.182	
Nodular sclerosis	223 (10; 202–243)		
Mixed cellularity	244(13; 218–269)		
Epstein–Barr virus-		0; 0.994	
positive	227 (15; 197–257)		
negative	236 (9; 217–256)		
Stage		0.267; 0.875	
I **	/		
II	217 (14; 190–245)		
III	198 (20; 158–238)		
IV	175 (17; 141–209)		
FOXP3		4.3; 0.039	2.3 (1–5); 0.046
≤327	219 (13; 193–246)		
>327 ^†^	247 (9; 229–246)		
CD8		1.5; 0.214	
≤435	217 (12; 194–240)		
>435	248 (11; 227–269)		
FOXP3/CD8		0.101; 0.750	
≤8	235 (12; 211–258)		
>8	228 (11; 27–250)		
CD4		14.1; *p* < 0.001	5.5 (2–14.7); 0.001
≤1898	163 (12; 140–187)		
>1898 ^†^	267 (7; 253–272)		
FOXP3/CD4		2.6; 0.104	
≤263	223 (13; 196–249)		
>263	244 (10; 224–264)		

* LR, Log rank. Legend: HR, hazard ratio; CI, confidence interval; ^†^ reference level, ** not a single relapse

**Table 9:** Relation of relapse to overall survival of patients with classic Hodgkin lymphoma.

		Overall Survival (Months)			
Relapse	N	Mean (SE)	95% CI	LR; *p* *	DIED
no	96	241 (6.4)	22.8–253	26.6; <0.001	17
yes	28	119 (16)	87–151		11
unknown	35	198 (16.5)	165–230		18

* LR, Log rank; SE, standard error; CI, confidence interval.

## Data Availability

All data are available upon request from the corresponding author.
